# The role of the *Pseudomonas aeruginosa* hypermutator phenotype on the shift from acute to chronic virulence during respiratory infection

**DOI:** 10.3389/fcimb.2022.943346

**Published:** 2022-07-22

**Authors:** Kalen M. Hall, Zachary F. Pursell, Lisa A. Morici

**Affiliations:** ^1^ Department of Microbiology and Immunology, Tulane University School of Medicine, New Orleans, LA, United States; ^2^ Department of Biochemistry and Molecular Biology, Tulane University School of Medicine, New Orleans, LA, United States

**Keywords:** *Pseudomonas*, hypermutation, mismatch repair, microevolution, chronic respiratoryinfection, virulence

## Abstract

Chronic respiratory infection (CRI) with *Pseudomonas aeruginosa* (Pa) presents many unique challenges that complicate treatment. One notable challenge is the hypermutator phenotype which is present in up to 60% of sampled CRI patient isolates. Hypermutation can be caused by deactivating mutations in DNA mismatch repair (MMR) genes including *mutS*, *mutL*, and *uvrD*. *In vitro* and *in vivo* studies have demonstrated hypermutator strains to be less virulent than wild-type Pa. However, patients colonized with hypermutators display poorer lung function and a higher incidence of treatment failure. Hypermutation and MMR-deficiency create increased genetic diversity and population heterogeneity due to elevated mutation rates. MMR-deficient strains demonstrate higher rates of mucoidy, a hallmark virulence determinant of Pa during CRI in cystic fibrosis patients. The mucoid phenotype results from simple sequence repeat mutations in the *mucA* gene made in the absence of functional MMR. Mutations in Pa are further increased in the absence of MMR, leading to microcolony biofilm formation, further lineage diversification, and population heterogeneity which enhance bacterial persistence and host immune evasion. Hypermutation facilitates the adaptation to the lung microenvironment, enabling survival among nutritional complexity and microaerobic or anaerobic conditions. Mutations in key acute-to-chronic virulence “switch” genes, such as *retS*, *bfmS*, and *ampR*, are also catalyzed by hypermutation. Consequently, strong positive selection for many loss-of-function pathoadaptive mutations is seen in hypermutators and enriched in genes such as *lasR*. This results in the characteristic loss of Pa acute infection virulence factors, including quorum sensing, flagellar motility, and type III secretion. Further study of the role of hypermutation on Pa chronic infection is needed to better inform treatment regimens against CRI with hypermutator strains.

## Introduction

*Pseudomonas aeruginosa* (Pa) is a ubiquitous Gram-negative bacterium known to cause a wide range of opportunistic infections, including respiratory, wound, urinary tract, surgical site, and bloodstream infections. Pa has been designated a global threat due to increasing rates of multidrug resistant nosocomial infections, but it has long been known as the primary cause of morbidity and mortality in cystic fibrosis (CF) patients (Rajan, 2002). CF is an autosomal recessive disorder caused by over 2,000 documented variants in the CF transmembrane conductance regulator (CFTR) gene, leading to multisystem pathology (Bergeron and Cantin, 2019). The disease affects over 30,000 people in the United States and has a poor prognosis with a median age of death of 34 (Cystic Fibrosis Foundation, 2021). In the lungs, altered CFTR function leads to thick mucosal secretions which create a unique hospitable environment for microbes (Ciofu et al., 2013). Patients become colonized in the respiratory tract in ages as early as 1 with bacteria such as Pa, *Staphylococcus aureus*, *Haemophilus influenzae*, and *Stenotrophomonas maltophilia*, but Pa predominates by age 18 (Rajan, 2002; Cystic Fibrosis Foundation, 2021). Chronic respiratory infection (CRI) with Pa leads to excessive inflammation and eventual tissue necrosis and lung failure (Ciofu et al., 2013).

CRI with Pa in the context of the CF lung poses many unique challenges for treatment. The infection is characterized by the Pa mucoid phenotype, high levels of drug resistance, persistence, and a shift from an acute to a chronic virulence state resulting in treatment failure. During CRI, Pa downregulates the virulence factors needed for establishment of acute infection, including LasR-mediated quorum sensing, type III secretion, twitching motility and adhesion mediated by flagella and pili. Instead, Pa expresses factors that favor persistence in the CF lung such as alginate overproduction, biofilm formation, and alternate metabolic pathways (Smith et al., 2006; Bragonzi et al., 2009). In the chronic virulence state, alginate overproduction, or “mucoidy”, in particular leads to greater regional lung inflammation and impairs both host immune defenses and therapeutic treatments (Malhotra et al., 2019).

Another challenge is the high prevalence (up to 60% of sampled patients) of hypermutator (defined as having 20-fold higher mutants per total viable cells than wild-type) strains in chronic Pa CF lung infections, which are overwhelmingly caused by deactivating mutations in the DNA mismatch repair (MMR) genes such as *mutS*, *mutL*, and *uvrD* (**Table 1**) (Oliver et al., 2000; Oliver et al., 2002a; Hogardt et al., 2006). Hypermutator isolates deficient in the GO system are very rarely observed, but most isolates to be complemented with GO system genes such as *mutT* or *mutM* (Oliver et al., 2002b) The bacterial DNA MMR system is responsible for repairing replicative insertion or slippage errors that were not corrected by DNA polymerase proof-reading activity (Kunkel and Erie, 2005; Iyer et al., 2006). In the absence of MMR, mutations are biased towards T>C and C>T transitions and insertion-deletions (indels) in homopolymers, implicating genes containing these sequences as mutational hotspots in hypermutators (Lee et al., 2012; Marvig et al., 2013). Hypermutators are thought to be so prevalent in CRI because of the positive co-selection of the resulting adaptive mutations consequent of MMR-deficiency (Gutiérrez et al., 2004). Induction of the hypermutator phenotype has been associated with chronic oxidative stress and with chronic antibiotic treatment (Ciofu et al., 2005; Dößelmann et al., 2017). Hypermutators appear to be associated with chronicity of infection, as none were found until 5 years after the onset of infection in a sample of CF isolates and are incredibly rare in acute infection isolates (<1%) (Gutiérrez et al., 2004; Ciofu et al., 2005). In addition, patients colonized with hypermutators are reported to have poorer patient lung function measured *via* percent forced expiratory volume and mean forced expiratory volume per forced vital capacity (Waine et al., 2008; Ferroni et al., 2009). Colonization with hypermutators is also associated with greater instance of multidrug resistance and treatment failure (Macía et al., 2005).

**Table 1:** Pa MMR genes and their respective functions, along with common point mutation positions resulting in protein inactivation (Oliver et al., 2002a; Hogardt et al., 2006; On and Welch, 2021).

Gene	Product function	Conserved residues resulting in loss-of-function
*mutS*	DNA mismatch/short indel recognition	A187, F653, R842, K852
*mutL*	Endonuclease, nicks daughter strand (methylation-independent)	K307
*uvrD*	DNA helicase, unwinds double helix to allow for damage removal	A31, G32, G36

Despite these trends in clinical data, *mutS*-knockout strains of Pa are outcompeted by wild-type *in vitro* and *in vivo* murine models. Strains deficient in MutS also display attenuated virulence and reduced capacity for colonization (Mena et al., 2007; Montanari et al., 2007). Clinical data has long suggested the independent emergence of hypermutators in CF patients, but recent phylogenetic analyses of widespread clonal lineages of Pa CF isolates demonstrated evidence for interpatient transmission (Oliver and Mena, 2010; López-Causapé et al., 2017). The discrepancy between clinical and experimental observations suggests that the hypermutator phenotype may be critical for bacterial adaption or survival during CRI. High mutation rates have been shown to be beneficial in early colonization and mutator alleles can become fixed in a fraction of the population due to their evolutionary advantage, even though randomly occurring deleterious mutations at secondary sites can be disadvantageous (Taddei et al., 1997; Giraud et al., 2001). Hypermutation has also been observed in *S. aureus*, *H. influenzae*, *Escherichia coli*, *Salmonella enterica* and *Neisseria meningitidis*, possibly implicating it as a conserved mechanism for rapid adaptation (LeClerc et al., 1996; Matic et al., 1997; Sniegowski et al., 1997; Richardson et al., 2002; Prunier et al., 2003; Román et al., 2004).

**Figure 1:**
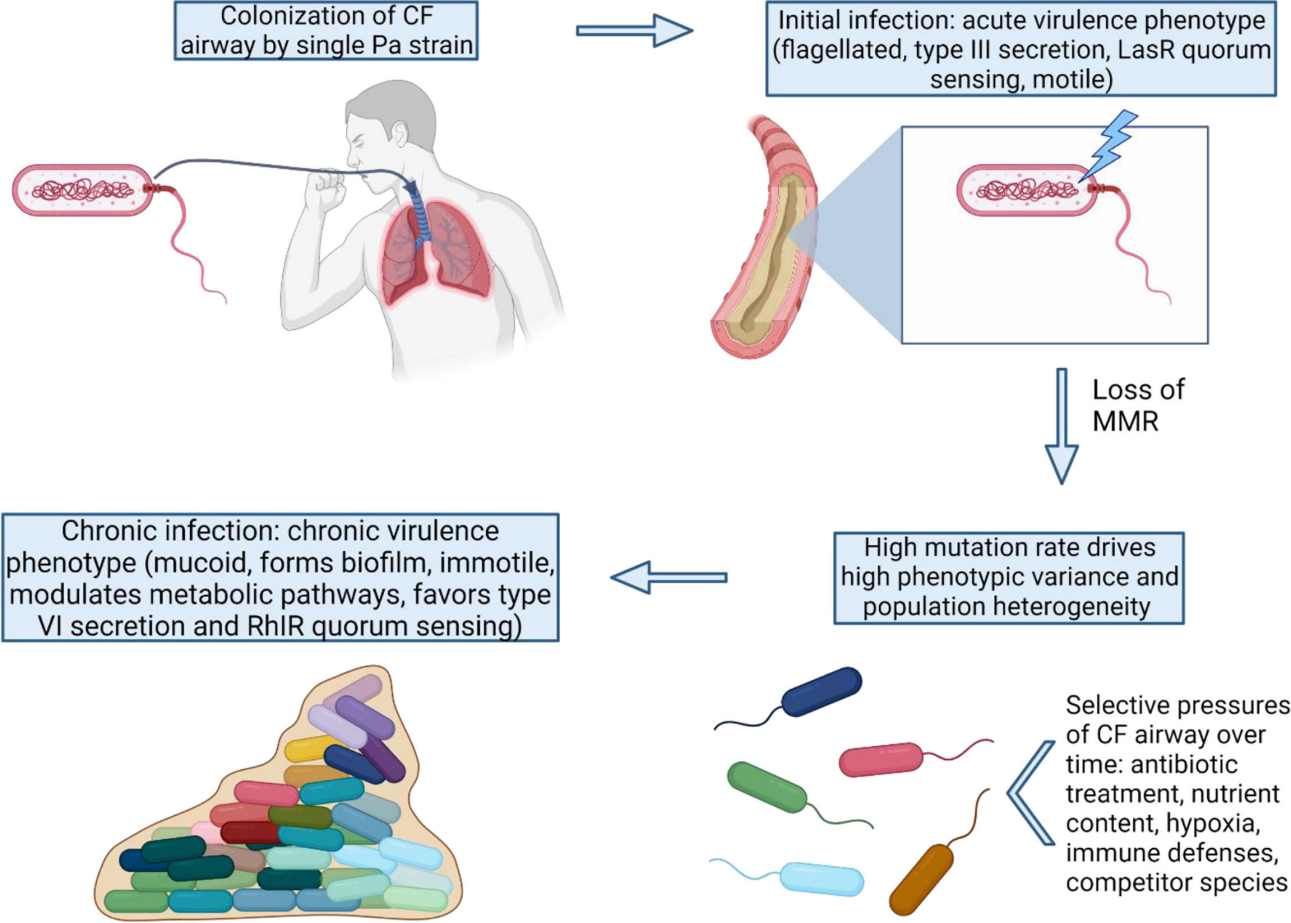
MMR-deficiency catalyzes the shift from an acute to chronic P. aeruginosa virulence state. Once MMR is lost in the initial colonizing strain, many adaptive pathways can be exploited by Pa *via* high mutation rates. The variants with mutations favoring the chronic virulence state confer advantages for long-term survival and persistence under the strong selective pressures of the CF lung.

In this review, we examine the role of the Pa hypermutator phenotype (caused by MMR-deficiency) found in CRI in the shift from the acute to chronic virulence state (summarized in **Figure 1**). We will describe how hypermutation allows for genetic population heterogeneity and phenotypic diversity and how MMR-deficiency catalyzes the establishment of the most common mutant *mucA22* allele causing mucoidy (Moyano et al., 2007). Hypermutation allows for the rapid adaptation to the CF lung microenvironment *via* mutations in metabolic pathways allowing for survival in the high amino acid content and microaerobic conditions. In addition, mutations in master transcriptional regulators act as ‘switches’ that shift Pa from the acute to the chronic virulence state. Hypermutators are strongly associated with pathoadaptive loss-of-function mutations in acute virulence genes that contribute to the transition of virulence state as well.

## Hypermutation creating population heterogeneity and phenotypic diversity

A hallmark of Pa CRI is a phenomenon known as adaptive radiation that contributes to Pa persistence in the CF lung. Due to its large genome size (>6 Mb) and sophisticated networks of transcriptional regulation, a single parent Pa strain has the capacity to occupy specific environmental niches *via* divergence into adapted sublineages (Stover et al., 2000; Klockgether et al., 2011; Markussen et al., 2014). These sublineages differ at the genomic and phenotypic levels but coexist creating a heterogenous population (Chung et al., 2012; la Rosa et al., 2018). Nutritional complexity and high mucin levels in CF airways alone are sufficient to drive Pa’s divergence into sublineages and phenotypic diversity, but this diversification is even further enhanced by host immune pressure and competitor microbial species (Schick and Kassen, 2018; la Rosa et al., 2019). Whole genome sequencing of isolates derived from CF patients has revealed that an initial colonizing strain of Pa undergoes a period of rapid adaptation followed by a long period of genetic drift with minor changes. This is seen in non-mutator Pa populations in CF airways with a reported mutation rate of 7.2 x 10^-11^ single nucleotide polymorphisms (SNPs) per base pair (bp) per generation (Yang et al., 2011).

Hypermutation creates greater genetic diversity in a Pa population, allowing for further phenotypic diversity and driving evolution (Mena et al., 2008). Laboratory evolution experiments with a MutS-deficient Pa strain showed significantly greater diversification of colony morphology that demonstrate increased antibiotic resistance and decreased cytotoxicity similar to CF isolates (Smania et al., 2004). Hypermutation affecting genetic and phenotypic diversity is observed extensively in clinical isolates. MutS-deficient paired isolates differed by 344 SNPs and 93 indels, compared to 54 SNPs/38 indels and 1 SNP/8 indels of two pairs of wild-type isolates from different patients (Chung et al., 2012). A longitudinal genetic analysis of 13 isolates from an Argentinian patient and 14 isolates from a Danish patient revealed sublineages with extensive intra-patient genomic diversity (Feliziani et al., 2014). Hypermutators comprised 90% of the heterogenous population of isolates from both the Argentinian and Danish patient, indicating that they dominate and outcompete non-mutator isolates under CF airway selective pressures. The reported *in vivo* mutation rate of the populations was 100 SNPs/year, which is 40-fold higher than non-mutator isolates. Characteristic of adaptive radiation, genomic variation showed coexistence of equally fit subpopulations that arose from multiple evolutionary events. Parallel convergent evolution across sublineages and patient populations indicate hypermutation can target genes that optimize fitness in the CF airway independent of geography (Feliziani et al., 2014). A retrospective study of the DK2 clone type (a lineage of Pa strains causing chronic infection in Danish CF patients) and its transmission events and subsequent divergence into intra-patient sublineages also demonstrated parallel evolution in genes related to antibiotic resistance, regulatory functions, and the cell envelope, which is thought to play a role in immune evasion. Many of these genes contained homopolymers, which are known MMR-deficiency mutation hotspots, and the number of mutations accumulated correlated with homopolymer run length (Marvig et al., 2013). This has been expanded upon with another analysis of longitudinally collected CF isolates containing many hypermutators that showed parallel convergent evolution in genes involved in central metabolism and virulence factors (Marvig et al., 2015).

## The shift to mucoidy and biofilm development

Mucoidy is a unique characteristic of CRIs with Pa and is caused by the overproduction of the exopolysaccharide alginate. Pa’s conversion to mucoidy during CRI is mostly caused by inactivating mutations in *mucA* (~85%), a negative regulator of sigma factor *algU*. Disruption of MucA leads to constitutive expression of AlgU and alginate biosynthesis (Martin et al., 1993; Boucher et al., 1997). The mucoid phenotype is highly virulent and is associated with chronic infections, increased inflammation, and increased patient mortality (Malhotra et al., 2019). Alginate overproduction aids Pa in evading macrophage killing *via* scavenging of free radicals (Simpson et al., 1989). Alginate also interferes with antibody-independent opsonic killing and Th1-mediated killing (Pier et al., 2001). Alginate expression is associated with increased pro-inflammatory cytokines that exacerbate tissue damage and pathology (Song et al., 2003).

Pa mucoid isolates are more likely to be hypermutators than non-mucoid isolates, demonstrating an association between the two phenotypes (Waine et al., 2008). This could be because both phenotypes are associated with chronic infection. However, *in vitro* data suggests that hypermutation could be driving mucoid conversion. A MutS-deficient Pa strain showed significantly increased emergence of mucoid mutants when cultured *in vitro* compared to wild-type. This was associated with a single base deletion in a run of five consecutive guanines (G5-SSR426). This deletion causes a frameshift and results in an inactivated mutant allele (*mucA2*2), which is observed in up to 40% of mucoid CF isolates (Bragonzi et al., 2006; Moyano et al., 2007). A forward mutation model of *mucA* demonstrated a critical role of G5-SSR426 in mucoid conversion in MMR-deficient cells and emphasized homopolymers as a main target for hypermutators (Moyano and Smania, 2009). It is important to note that *mucA* mutations have been found to occur prior to MMR-inactivating mutations, and that no statistically significant link could be established between hypermutability and *mucA* mutations in two studies: one assessing 70 samples from 10 CF patients and another with 38 isolates from 26 CF patients (Ciofu et al., 2010; Feliziani et al., 2010). Together, these data suggest that hypermutation is not a prerequisite for mucoidy but may drive conversion when present.

Alginate overproduction also plays a key role in Pa biofilm formation and architecture (Nivens et al., 2001; Ghafoor et al., 2011). In addition to mucoid conversion, hypermutators also show high rates of missense mutations in *bfmS*, a sensor histidine kinase that negatively regulates *bfmR* which is responsible for biofilm maturation (Cao et al., 2020). Biofilms display increased resistance to antibiotics and phagocytosis and worsen inflammation during CRI. Biofilms also display increased mutagenesis, promoting adaptation to the CF lung (Høiby et al., 2010). However, sequencing of 12 isolates of the DK2 lineage, all deemed to be non-mutators, showed no increase in mutation rate in biofilms (Yang et al., 2011). Mutagenesis data obtained *in situ* with biofilms implicates the importance of microcolonies. MMR-deficient Pa showed enhanced microcolony formation and growth, and the mutation rates in the microcolonies were elevated compared to planktonic forms (Conibear et al., 2009). This implicates hypermutability in Pa’s exceptional capacity to adapt as a biofilm.

## The adaptation to the CF lung microenvironment

### Hypermutation aids in survival in complex nutritional environment

The CF lung poses a unique and complex environment in terms of bacterial nutrient uptake and survival as it is comprised of high amounts of mucin, lipids, and amino acids (Thomas et al., 2000). Thick mucosal secretions also create pockets of hypoxia within the airways, creating unusual microaerobic to anaerobic bacterial growth conditions (Worlitzsch et al., 2002). A longitudinal study of sequential isolates of the DK2 clone family revealed oxygen metabolism as a hotspot for adaptive evolution (la Rosa et al., 2018). A transcriptomic and proteomic analysis of 13 sequential isolates including both MMR-deficient hypermutators and MMR-intact non-mutators revealed key metabolic adaptation catalyzed by hypermutation. Transcripts of genes involved in the anaerobic arginine deaminase pathway, such as *oprF*, *azu*, *ccpR*, *aotJ*, and *braC*, were increased in mutators. This pathway allows for production of adenine triphosphate using amino acid arginine under low oxygen conditions, suggesting the hypermutators were well adapted to the rich amino acid content and hypoxia in the CF lung. Interestingly, OprF has been implicated in biofilm viability in anaerobic environments, and Azu and CcpR protect against reactive nitrogen species released as byproducts of anaerobic respiration (Foote et al., 1992; Hassett et al., 2002; Yoon et al., 2002). Expression of genes in the arginine succinyltransferase pathway, which converts arginine into TCA cycle intermediates, was also increased in hypermutators. Genes needed for lipid metabolism (PA2886-93, *foaAB*, *acpP*, *accB*, and *fabI*) were also highly up-regulated in hypermutators. Gene expression profiles differed between hypermutators but showed a signature of convergent parallel evolution on these gene sets, suggesting adaptive evolution (Hoboth et al., 2009). Adaptive laboratory evolution of a hypermutator CF isolate showed overexpression of *nos*, *nor*, and *nir* operons due to RpoN overexpression which can mitigate toxic effects of anaerobic respiration as a vital adaptive event. Reversion to acute phenotype during laboratory evolution also showed upregulation in *cioA* and *cioB* needed for aerobic respiration (la Rosa et al., 2021). Genomic analysis of longitudinal hypermutator isolates showed parallel reductive evolution in catabolism pathways, resulting in a high number of auxotrophies, thought to serve as an energy conservation mechanism due to the rich amino acid environment in the CF airway (Feliziani et al., 2014).

### Hypermutation catalyzes mutagenesis in master transcriptional regulators

Pa employs a large arsenal of virulence factors that are tightly controlled by a complex network of transcription factors to minimize unnecessary energy expenditure (Balasubramanian et al., 2013). Transcriptional plasticity has been implicated in the flexibility of Pa to occupy many environmental niches and to persist in chronic CF lung infections (Rossi et al., 2018). Hypermutator strains have been documented to have many nonsynonymous mutations in master transcription regulators that mediate the switch from an acute to chronic virulence state. Inactivating mutations in *lasR* (a master quorum sensing regulator) are highly correlated with hypermutability in CF isolates (Whiteley et al., 1999; Bjarnsholt et al., 2010). LasR mutants are associated with chronic infection and poorer patient outcome similar to hypermutability (Hoffman et al., 2009). MutS-deficient Pa displayed significant emergence of LasR-mutants caused by indel frameshifts compared to wild-type *in vitro* (Luján et al., 2007). It is important to note that, like MucA, LasR mutants have been observed before MMR-deficiency, so it is not a requirement (Ciofu et al., 2010).

Additional common loci for mutation in hypermutator strains are *gacS* and *retS* that regulate the Gac/Rsm signaling pathway. GacS negatively regulates the pathway and promotes expression of type VI secretion and Pel polysaccharides. Through upregulation of small regulatory RNAs RsmY and RsmZ, GacS activity also downregulates type III secretion (Sall et al., 2014; Valentini et al., 2018). Through these pathways, GacS promotes characteristics of the chronic virulence state. RetS activity attenuates GacS signaling and therefore promotes expression of factors of the acute virulence state (Francis et al., 2018). RetS has been found to be a hotspot for loss-of-function frameshifts in hypermutators. Interestingly, GacS and GacA (the other member of the GacS/GacA two-component system controlling RsmY and RsmZ expression) have also been identified as hotspots for convergent evolution in hypermutators (Feliziani et al., 2014; Marvig et al., 2015; la Rosa et al., 2021). GacA mutants have been associated with nitrogen metabolism upregulation, type VI secretion, and reduced motility (Wei et al., 2013; Huang et al., 2019). GacS mutants appear to confer an evolutionary advantage in the CF airway through formation of small colony variants in biofilms with increased resistance to immune defense and antibiotics (Davies et al., 2007; Nelson et al., 2010). Large numbers of AmpR and ExsA mutants have also been observed in hypermutators with adaptive consequences in type III secretion system functions, quorum sensing, immune evasion, and nitrogen metabolism (Balasubramanian et al., 2014; Marvig et al., 2015; Huang et al., 2019; Tian et al., 2019; la Rosa et al., 2021).

## Pathoadaptive loss of function mutations in key acute virulence genes

Numerous genetic analyses of longitudinal CF isolates have revealed overwhelmingly large numbers of mutations in key Pa virulence genes and have consistently showed a strong signature of purifying selection for these loss-of-function pathoadaptive mutations (Smith et al., 2006; Chung et al., 2012; Feliziani et al., 2014; Marvig et al., 2015; Wee et al., 2018). One example is the loss of quorum sensing in CRI with Pa due to LasR loss-of-function. However, recent evidence has shown that quorum sensing may not be lost, but hypermutation helps rewire it to favor the LasR-independent RhII-RhIR alternate pathway (Feltner et al., 2016; Chen et al., 2019; Kostylev et al., 2019). RhIR mutants are highly correlated with hypermutability in CF isolates (Bjarnsholt et al., 2010). *In vitro*, RhIR mutants were not found with evolution of a MutS-deficient Pa strain, whereas LasR mutants did emerge, suggesting differing selective pressures on the two pathways (Luján et al., 2007). In the context of Pa CRI, RhII has also been associated with anaerobic biofilm viability, which quorum sensing plays an especially important role in due to close spatial arrangement and population heterogeneity (Hassett et al., 2002; Darch et al., 2018).

Another common pathoadaptive mutation in CF isolates is the loss of type III secretion. This usually results from mutations in transcription regulators such as *retS* and *exsA*, as mentioned previously. Hypermutators show significantly more downregulation of genes involved in type III secretion compared to non-mutators, and they do not produce the major type III secretion product ExoS (Hoboth et al., 2009). Instead, adaptive laboratory evolution with a hypermutator CF isolate shows that shift in expression of type III to type VI secretion is a key adaptive event (Moscoso et al., 2011; la Rosa et al., 2021). Hypermutators also show significantly more downregulation in flagellar proteins compared to non-mutators, and show convergent evolution in *flgG* and *fliD*, associated with the chronic virulence state of loss of motility (Hoboth et al., 2009; la Rosa et al., 2021). This is recapitulated *in vitro*, as MutS-deficient Pa shows impaired swimming and twitching motility, suggesting the switch to favor biofilm formation (Smania et al., 2004). The genes targeted for pathoadaptive mutations in hypermutators are summarized in **Table 2**.

**Table 2:** Summary of genes targeted for convergent evolution in the CF lung during CRI with Pa, catalyzed by hypermutation.

Gene	Product function	Type of pathoadaptive mutation	Downstream phenotypic result
*mucA*	Negative regulator of *algU*	Loss-of-function	Alginate overproduction, mucoidy
*bfmS*	Sensor histidine kinase, negative regulator of *bfmR*	Loss-of-function	Biofilm maturation
*lasR*	Master transcriptional regulator	Loss-of-function	Loss of LasI-LasR quorum sensing network
*gacS*	Negative regulator of Gac/Rsm signaling	Loss-of-function	Formation of SCVs in biofilms
*gacA*	Regulator of RsmY and RsmZ expression	Loss-of-function	Upregulation of nitrogen metabolism, type VI secretion, and reduced motility
*retS*	Attenuates GacS signaling in Gac/Rsm pathway	Loss-of-function	Upregulation of type VI secretion and Pel polysaccharides
*ampR*	Master transcriptional regulator	Loss-of-function	Promotes type VI secretion, affects Gac/Rsm, quorum sensing, adhesion
*exsA*	Master transcriptional regulator	Loss-of-function	Affects quorum sensing, nitrogen metabolism
*flgD*	Flagellar protein	Loss-of-function	Loss of motility and twitching
*fliD*	Flagellar cap protein	Loss-of-function	Loss of adhesion

## Conclusions and perspectives

Hypermutators play a vital role in the survival and persistence of Pa in CRI by allowing rapid diversification and adaptation to the CF lung environment. This results in the shift from the acute virulence phenotypes (type III secretion, motility, toxin production) to the chronic virulence phenotypes. Isolates having undergone the shift to a chronic virulence state display mucoidy, biofilm formation, modulation of metabolic pathways, alteration of quorum sensing, type VI secretion, and loss of motility. The high prevalence of hypermutators arising in the CF lung underscores the need for the adaptability afforded by genetically diverse isolates. This mutagenesis is preferred despite the simultaneous increased probability of accumulating deleterious mutations and potential reduced virulence.

This review reveals many discrepancies between longitudinal genetic analyses and *in vitro* adaptive evolution, notably in data concerning the effect of hypermutability on MucA and LasR. Although mutation rate and accumulation of mutations is higher in hypermutators, numbers of variants in target genes are usually not significantly different between hypermutator and non-mutator CF isolates (Mena et al., 2008). This suggests that hypermutation may not affect a specific adaptive trait significantly over the course of CRI, but rather has a generalized effect of facilitating adaptive evolution. As the selective pressures of the CF lung are the same on both hypermutators and non-mutators, it is reasonable for both to undergo similar genetic and phenotypic changes just at different rates. In fact, the only trait significantly linked to hypermutation is antibiotic resistance (Macía et al., 2005; Feliziani et al., 2010). This could be due to the large bottlenecking effect of antibiotic treatment on a population (Windels et al., 2021).

Although this review mainly addresses the role of hypermutation in the adaption of Pa to the CF lung environment, hypermutation has also been shown to play a role in adaptation in other disease states as well. PAHM4 (a bronchiectasis Pa isolate) displays *mutS* inactivating alleles similar to CF isolates (Warren et al., 2011). It also contains *mucA22* causing mucoidy, likely caused by MutS-deficiency as discussed above. The isolate shows similar characteristics of loss of motility and type III secretion and demonstrates high levels of antibiotic resistance (Varga et al., 2015). Similar to CF, bronchiectasis airways are known for having high mucin levels, altered nutritional complexity, and a widely diverse diseased-state lung microbiome, so perhaps similar selective pressures are driving convergence on these highlighted similarities (H. Richardson et al., 2019).

The prevalence of the hypermutator phenotype in Pa CRI and its prominent role in adaptation challenges the common assumption in microbiology that strains and isolates are clonal and can be treated as such. Population heterogeneity is overwhelmingly seen in CF isolates. With the occurrence of hypermutation in other diseases and even other species, it is possible that many other bacterial isolates also display high levels of population heterogeneity. Hypermutator *S. aureus* and *H. influenzae* isolates have been documented in CF patients and are associated with higher rates of antibiotic resistance, similar to Pa (Prunier et al., 2003; Román et al., 2004). How the hypermutator phenotype affects interspecies competition in the context of the CF lung is yet to be understood. As mentioned previously, hypermutability also occurs in pathogenic *E. coli*, *S. enterica*, and *N. meningitidis* (LeClerc et al., 1996; Matic et al., 1997; A. R. Richardson et al., 2002). It would be interesting to explore the role of hypermutation in different species and disease contexts and to see if it similarly drives adaptation to the host environment fostering survival. Hypermutation and its effect on bacterial pathogenesis poses many interesting questions for future study.

## Author contributions

KH performed the literature review and drafted the manuscript. LM and ZP revised the manuscript and provided supervision. All authors contributed to the article and approved the submitted version.

## Conflict of interest

The authors declare that the research was conducted in the absence of any commercial or financial relationships that could be construed as a potential conflict of interest.

## Publisher’s note

All claims expressed in this article are solely those of the authors and do not necessarily represent those of their affiliated organizations, or those of the publisher, the editors and the reviewers. Any product that may be evaluated in this article, or claim that may be made by its manufacturer, is not guaranteed or endorsed by the publisher.
